# Generating Evidence to Improve the Response to Neglected Diseases: How Operational Research in a Médecins Sans Frontières Buruli Ulcer Treatment Programme Informed International Management Guidance

**DOI:** 10.1371/journal.pntd.0004075

**Published:** 2015-11-12

**Authors:** Daniel P. O’Brien, Nathan Ford, Marco Vitoria, Kingsley Asiedu, Alexandra Calmy, Philipp Du Cros, Eric Comte, Vanessa Christinet

**Affiliations:** 1 Manson Unit, Médecins Sans Frontières, London, United Kingdom; 2 Department of Infectious Diseases, Barwon Health, Geelong, Australia; 3 Department of Medicine and Infectious Diseases, Royal Melbourne Hospital, University of Melbourne, Melbourne, Australia; 4 HIV Department, World Health Organization, Geneva, Switzerland; 5 Department of Control of Neglected Diseases, World Health Organization, Geneva, Switzerland; 6 Department of HIV, University Hospitals of Geneva, Geneva, Switzerland; 7 Medical Unit, Médecins Sans Frontières, Geneva, Switzerland; Fondation Raoul Follereau, FRANCE

## Introduction

Neglected Tropical Diseases (NTDs) are estimated to cause more than 500,000 deaths per year, almost exclusively affecting those living in impoverished rural and urban areas of low-income countries [1]. They are also characterised by a lack of research support and development of specific drugs, diagnostics, and vaccines [2,3]. For many NTDs, scientific information is lacking due to a paucity of research, often because they occur in settings with little access to the resources required to conduct high-quality research such as randomised trials. This lack of research in turn limits the development of evidence-based guidance to inform clinical and programme responses. Humanitarian organisations often work in these settings and commonly have access to the extra resources and collaborations with academic institutions needed to support such activities. Therefore, these organisations have a relatively unique opportunity to obtain important, new information on NTDs through the performance and publication of operational research using observational data. While observational studies cannot be used to make causal claims due to the inherent risk of bias and confounding, such data can nevertheless provide valuable information for health care management, hypothesis generation, and trend analysis [4]. Here we describe how operational data generated from a Médecins Sans Frontières (MSF) Buruli ulcer (BU) management programme provided important information that contributed to the development of a global BU treatment guidance that addressed a number of complex management issues involving BU-HIV coinfection.

BU is a necrotising infection of skin and subcutaneous tissue caused by *Mycobacterium ulcerans*. It commonly affects children in remote, resource-limited settings and, when severe, is associated with prolonged illness and long-term disability [5]. The main burden of BU is in West and Central Africa—regions also burdened with high HIV prevalence. All 15 countries in West and Central Africa reporting BU cases have an adult HIV prevalence of 1%–5%. Therefore, there is a significant potential for BU and HIV to occur in the same individual.

Until recently, however, there has been very little known about the epidemiology, clinical consequences, and management implications of BU-HIV coinfection. Critical unanswered questions include the following: Is HIV a risk factor for BU? Does HIV affect BU disease presentation and severity, or does it influence outcomes such as mortality, BU cure rates, and healing times? How should BU-HIV coinfected patients be managed, when should antiretroviral treatment (ART) start, and what BU treatment regimens should be used in the face of potential interactions with antiretroviral drugs?

In 2013, due to an increasing recognition of the importance and complexity of managing BU-HIV co-infection and the lack of guidance to aid in its management, World Health Organization (WHO) initiated the process of developing some core guidance principles, informed by a panel of experts [6,7]. Recommendations were based on their experience managing BU-HIV coinfection and what little scientific information was available on BU-HIV coinfection.

The main body of available published information from which to draw guidance came from the Médecins Sans Frontières BU treatment programme in Akonolinga, a town lying in the Nyong River basin in the central province of Cameroon [8]. The programme was based in a Ministry of Health district hospital and began treating BU patients in 2002. From the outset, a prospective observational database of routinely collected data was implemented for all patients treated for BU. HIV testing was initially introduced in 2002 for cases where clinical suspicion was high. However, it began to be recognised that BU-HIV coinfection was a significant issue, and in 2008 systematic HIV testing was introduced. Thus, the opportunity arose to acquire unique data on this coinfection. By May 2013, 1,130 patients had been treated for BU, and since the introduction of systematic HIV testing 29% of adults and 4% of children tested positive.

## Programmatic Evidence

MSF observational data were analysed to acquire information on a number of issues. Firstly, was there any evidence that HIV prevalence was increased in BU patients? The measured HIV prevalence in adult women with BU was about four times higher than the estimated regional prevalence (36% compared to 8%); in adult men with BU, HIV prevalence was about three times higher (17% compared to 5%); and in children, about eight times higher (4% compared to 0.7%) [8]. These data suggested that HIV infection may be higher in BU patients, supporting similar findings from the few other studies available [9,10]. This implied that BU patient populations should be considered a group with a higher prevalence of HIV infection, like those with tuberculosis, sexually transmitted infections, or malnutrition, in which comprehensive HIV testing may efficiently target previously undiagnosed HIV-positive patients and also allow better BU care for those who are HIV-infected.

**Fig 1:**
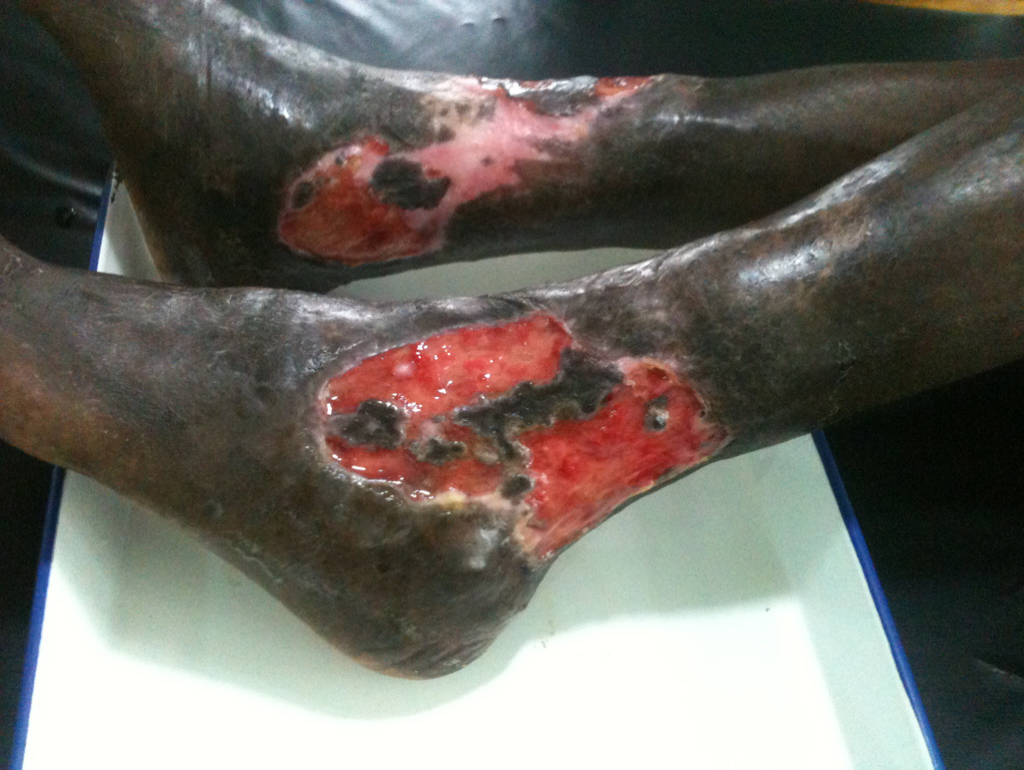
Image illustrating the severity of lesions in Buruli ulcer-HIV coinfected individuals; extensive bilateral ankle ulcers.

**Fig 2:**
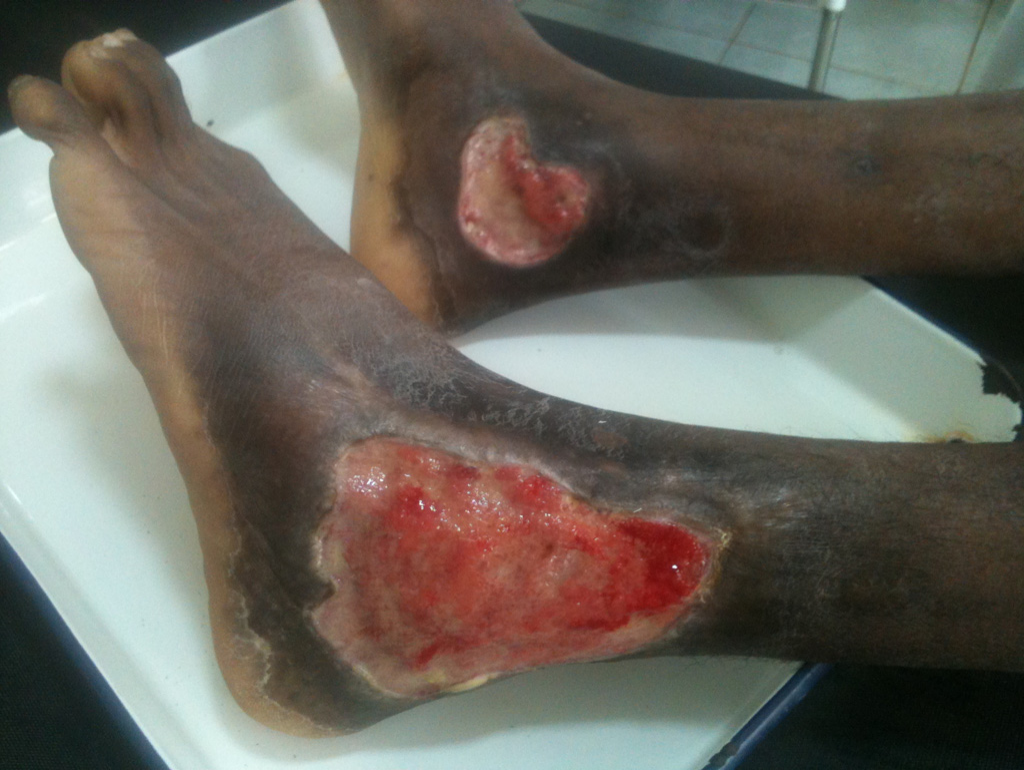
Image illustrating the severity of lesions in Buruli ulcer-HIV coinfected individuals; extensive bilateral ankle ulcers.

**Fig 3:**
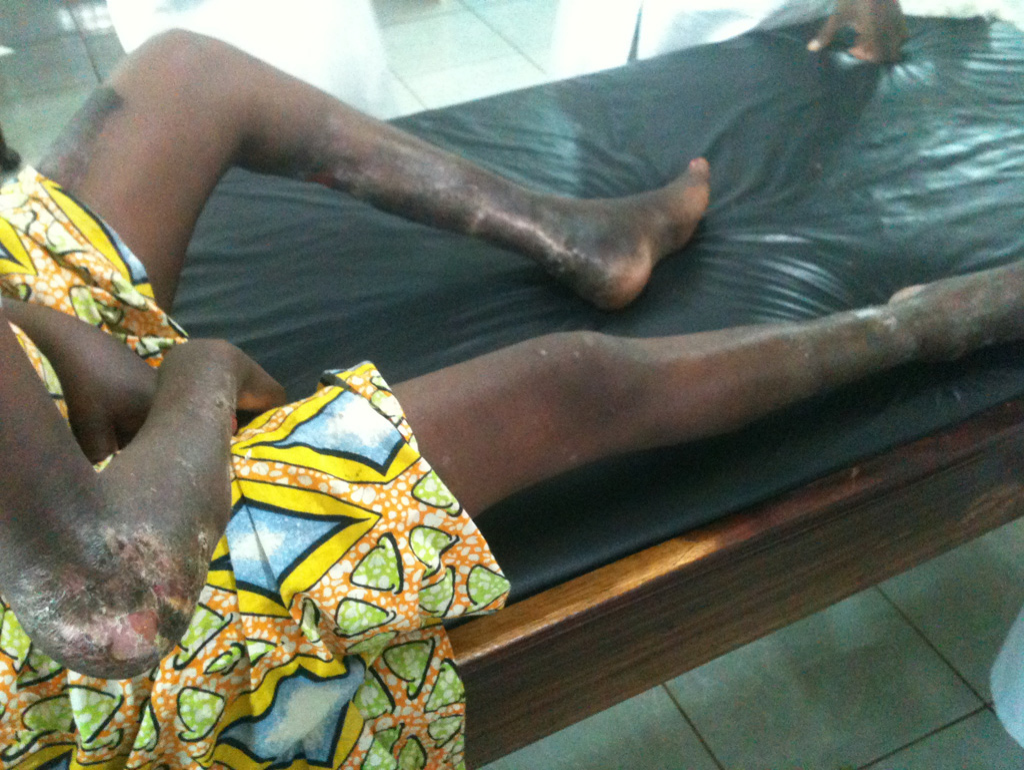
Image illustrating the severity of lesions in Buruli ulcer-HIV coinfected individuals; a woman with ulcers on her right elbow and both ankles.

Secondly, does HIV infection and its associated immune suppression affect the clinical presentation of BU disease? In comparing HIV-positive to HIV-negative BU patients presenting to Akonolinga hospital, multiple lesions were found to be about twice as common in HIV-positive patients (Figs 1–3). Furthermore, it was found that HIV-positive patients tended to have larger lesions, and a higher proportion had a more advanced form of BU (ulcerated compared with non-ulcerated lesions). Finally, it was found that for HIV-positive patients, the main lesion size increased with decreasing CD4 cell counts and, in the multivariable analysis, a level of ≤500 cells/mm^3^ was significantly associated with increased BU lesion size at baseline [8].

Hence, it appears from the Akonolinga data that HIV infection increases the severity of BU disease, and that disease severity worsens with increasing immune suppression. Apart from isolated case reports suggesting more severe disease in HIV-positive patients [11–15], this was the only data available reporting this clinical interaction.

Thirdly, does HIV infection affect BU outcomes? Most importantly, it was found in Akonolinga that the mortality rate was higher among HIV-positive BU patients compared to HIV-negative BU patients (11% dying compared to 1%). Furthermore, the median CD4 cell count amongst the eight HIV patients who died was relatively high, at 229 cells/mm^3^, and they died quite quickly, with a median time from BU diagnosis to death of only 41 days. Importantly, none were taking ART [8]. Hence, it appears from the Akonolinga data that mortality may be increased in BU-HIV coinfected patients, even in the absence of severe immune suppression. Once again, this was the only programme with published cohort data available on this issue, although some case reports existed of deaths in BU-HIV patients who had not commenced ART [11,12,16]. Furthermore, time to wound healing was twice as fast in those who had a CD4 count ≥500 cells/mm^3^ compared with <500 cells/mm^3^, suggesting that the level of immune suppression may influence the rate of BU healing [8].

There are many potential management implications of BU-HIV coinfection, including whether ART should be started in those being treated for BU and, if so, when. Significant potential benefits to ART exist, such as reduced opportunistic infections and mortality, especially at low CD4 cell counts [17]; improved BU outcomes such as increased cure rates, reduced recurrences, and shortened healing times; and potentially increased rates of retention in care. Potential risks include increased drug interactions and toxicity, reduced adherence rates due to high pill burdens, reduced effectiveness of BU treatments due to drug interactions, and increased incidence and severity of paradoxical reactions [7]. As described above, outcomes such as mortality and lesion healing may be influenced by HIV, but the expert panel needed further data to try and balance the risks and benefits of ART. Again, this was only available from the MSF programme in Akonolinga.

One of the important questions was whether BU-HIV patients are significantly immune-suppressed at BU diagnosis and thus in urgent need of ART. In Akonolinga this was the case, with 22% of those with a CD4 count performed having a level ≤200 cells/mm^3^ and a further 48% having a level between 201 and 500 cells/mm^3^. Hence, about two-thirds would qualify for ART, according to current WHO guidelines [8]. Furthermore, if CD4 cell count was not available, could the severity of BU lesions give a guide as to the level of immunosuppression and the need for ART? In the Akonolinga cohort, 80% of those with severe lesions (WHO category 2 or 3, a lesion ≥5 cm in diameter, or multiple lesions) had a CD4 cell count of <500 cells/mm^3^, compared with 55% of those with smaller lesions (WHO category 1, a lesion <5 cm in diameter) [8]. Hence, in the absence of CD4 cell counts, assessing the BU WHO category of disease could help target the patients with the highest likelihood of significant immune suppression and need for ART.

## Development of International Guidance

The expert panel was able to establish key principles for the management of HIV-BU coinfection, a number of which were significantly influenced by the MSF data described above [6,7]. Firstly, based on the fact that HIV prevalence appears to be increased in BU patients, and that it has significant clinical, management, and outcome implications, it was recommended that all BU patients should be offered quality, provider-initiated HIV testing and counseling. Secondly, based on a possible effect on outcomes like mortality and wound healing, ART should be initiated in all BU-HIV coinfected patients with symptomatic HIV disease (WHO clinical stage 3 or 4), regardless of CD4 cell count, and in those asymptomatic individuals with a CD4 count ≤500 cells/mm^3^, consistent with current WHO guidelines for all HIV-infected patients [17]. Thirdly, based on the MSF data that a high proportion of patients will be eligible for ART on standard CD4 criteria, if a CD4 count is not available, BU-HIV coinfected individuals with category 2 or 3 BU disease should be offered ART. However, due to a lack of proven benefit for those who are asymptomatic with a CD4 count >500 cells/mm^3^, and some potential risks, it was recommended that for asymptomatic patients with a CD4 count of >500 cells/mm^3^, ART should not commence until the CD4 count has fallen to or below 500 cells/mm^3^ or other criteria for ART have been met. Fourthly, weighing up the potential benefits regarding treatment outcomes, especially mortality, and that some may be influenced by the level of immune suppression, it was recommended that for eligible individuals ART should be commenced as soon as possible within eight weeks after commencing BU treatment, and as a priority in those with advanced HIV disease (CD4 count ≤350 cells/mm^3^ or WHO stage 3 or 4 disease), rather than deferring ART until completion of BU treatment.

## Conclusion

The development of international guidelines has evolved in recent years towards requiring recommendations to be based on evidence, ideally from randomized trials [18]. However, neglected tropical diseases continue to be left out of research efforts, with only 1% of registered clinical trials in 2012 directed at neglected diseases [3]. This creates a vicious circle in which, in the absence of evidence to inform guidance, there is no standardized management of neglected diseases, and little data collection to inform future guidance.

We have described our experience that illustrates how a humanitarian response to a neglected tropical disease, in this case MSF’s response to BU, can, through clinical practice and the study of observational data, despite its known limitations, allow the acquisition of knowledge that was important in informing international guidance for disease management. We hope that this experience will encourage other humanitarian organisations involved in providing medical assistance to populations affected by NTDs to prioritise operational research on these diseases, including their interaction with HIV, which for many could have a significant impact on their treatment and control [1,2].

## References

[1] Hotez PJ, Molyneux DH, Fenwick A, Ottesen E, Ehrlich Sachs S, et al (2006) Incorporating a rapid-impact package for neglected tropical diseases with programs for HIV/AIDS, tuberculosis, and malaria. PLoS Med 3: e102 16435908 10.1371/journal.pmed.0030102 PMC1351920

[2] Kappagoda S, Ioannidis JPA (2012) Neglected tropical diseases: survey and geometry of randomised evidence. Bmj 345: e6512–e6512. 10.1136/bmj.e6512 23089149 PMC3478233

[3] Pedrique B, Strub-Wourgaft N, Some C, Olliaro P, Trouiller P, et al (2013) The drug and vaccine landscape for neglected diseases (2000–11): a systematic assessment. Lancet Glob Health 1: e371–379. 10.1016/S2214-109X(13)70078-0 25104602

[4] Grimes DA, Schulz KF (2002) Descriptive studies: what they can and cannot do. Lancet 359: 145–149. 11809274 10.1016/S0140-6736(02)07373-7

[5] World Health Organisation (2012) Treatment of Mycobacterium ulcerans disease (Buruli ulcer): guidance for health workers. Geneva, Switzerland.

[6] World Health Organization (2014) Management of Buruli ulcer-HIV coinfection: technical update. Geneva.

[7] O'Brien DP, Ford N, Vitoria M, Christinet V, Comte E, et al (2014) Management of BU-HIV co-infection. Trop Med Int Health 19: 1040–1047. 10.1111/tmi.12342 24946829

[8] Christinet V, Rossel L, Serafini M, Delhumeau C, Odermatt P, et al (2014) Impact of HIV on the Severity of Buruli Ulcer Disease: Results from a Retrospective Study in Cameroon. Open Forum Infectious Diseases 1 (1). 10.1093/ofid/ofu021 PMC4324202 25734094

[9] Johnson RC, Nackers F, Glynn JR, de Biurrun Bakedano E, Zinsou C, et al (2008) Association of HIV infection and Mycobacterium ulcerans disease in Benin. Aids 22: 901–903. 10.1097/QAD.0b013e3282f7690a 18427211

[10] Yeboah-Manu D, Owusu-Mireku E, ruf MT, Aboagye S, Ampofo W, et al Buruli Ulcer and HIV Co-Infection in the Ga District of Ghana.; 2013; WHO Headquarters, Geneva, Switzerland.

[11] Johnson RC, Ifebe D, Hans-Moevi A, Kestens L, Houessou R, et al (2002) Disseminated Mycobacterium ulcerans disease in an HIV-positive patient: a case study. Aids 16: 1704–1705. 12172103 10.1097/00002030-200208160-00027

[12] Kibadi K, Colebunders R, Muyembe-Tamfum JJ, Meyers WM, Portaels F (2010) Buruli ulcer lesions in HIV-positive patient. Emerg Infect Dis 16: 738–739. 10.3201/eid1604.091343 20350411 PMC3321952

[13] Komenan K, Elidje EJ, Ildevert GP, Yao KI, Kanga K, et al (2013) Multifocal Buruli Ulcer Associated with Secondary Infection in HIV Positive Patient. Case Rep Med 2013: 348628 10.1155/2013/348628 24454398 PMC3886605

[14] Bayonne Manou LS, Portaels F, Eddyani M, Book AU, Vandelannoote K, et al (2013) Mycobacterium ulcerans disease (Buruli ulcer) in Gabon: 2005–2011. Me´decine et Sante´ Tropicales 23: 450–457. 10.1684/mst.2013.0259 24413612

[15] Toll A, Gallardo F, Ferran M, Gilaberte M, Iglesias M, et al (2005) Aggressive multifocal Buruli ulcer with associated osteomyelitis in an HIV-positive patient. Clin Exp Dermatol 30: 649–651. 16197379 10.1111/j.1365-2230.2005.01892.x

[16] Bafende AE, Lukanu NP, Numbi AN (2002) Buruli ulcer in an AIDS patient. S Afr Med J 92: 437 12146126

[17] World Health Organisation (2013) Consolidated Guidelines on the Use of Antiretroviral Drugs for Treating and Preventing HIV Infection: Recommendations for a Public Health Approach. Geneva, Switzerland.: World Health Organisation. 24716260

[18] Guyatt GH, Oxman AD, Vist GE, Kunz R, Falck-Ytter Y, et al (2008) GRADE: an emerging consensus on rating quality of evidence and strength of recommendations. Bmj 336: 924–926. 10.1136/bmj.39489.470347.AD 18436948 PMC2335261

